# Empirical evaluation of language modeling to ascertain cancer outcomes from clinical text reports

**DOI:** 10.1186/s12859-023-05439-1

**Published:** 2023-09-02

**Authors:** Haitham A. Elmarakeby, Pavel S. Trukhanov, Vidal M. Arroyo, Irbaz Bin Riaz, Deborah Schrag, Eliezer M. Van Allen, Kenneth L. Kehl

**Affiliations:** 1 Dana-Farber Cancer Institute, Boston, MA USA; 2 Al-Azhar University, Cairo, Egypt; 3 Harvard Medical School, Boston, MA USA; 4 The Broad Institute of MIT and Harvard, Cambridge, MA USA; 5 Stanford University, Stanford, CA USA; 6 Mayo Clinic, Rochester, MN USA; 7 Memorial-Sloan Kettering Cancer Center, New York, NY USA

**Keywords:** Cancer, Natural language processing, Clinical outcomes, Information extraction, Transformer-based language models

## Abstract

**Background:**

Longitudinal data on key cancer outcomes for clinical research, such as response to treatment and disease progression, are not captured in standard cancer registry reporting. Manual extraction of such outcomes from unstructured electronic health records is a slow, resource-intensive process. Natural language processing (NLP) methods can accelerate outcome annotation, but they require substantial labeled data. Transfer learning based on language modeling, particularly using the Transformer architecture, has achieved improvements in NLP performance. However, there has been no systematic evaluation of NLP model training strategies on the extraction of cancer outcomes from unstructured text.

**Results:**

We evaluated the performance of nine NLP models at the two tasks of identifying cancer response and cancer progression within imaging reports at a single academic center among patients with non-small cell lung cancer. We trained the classification models under different conditions, including training sample size, classification architecture, and language model pre-training. The training involved a labeled dataset of 14,218 imaging reports for 1112 patients with lung cancer. A subset of models was based on a pre-trained language model, DFCI-ImagingBERT, created by further pre-training a BERT-based model using an unlabeled dataset of 662,579 reports from 27,483 patients with cancer from our center. A classifier based on our DFCI-ImagingBERT, trained on more than 200 patients, achieved the best results in most experiments; however, these results were marginally better than simpler “bag of words” or convolutional neural network models.

**Conclusion:**

When developing AI models to extract outcomes from imaging reports for clinical cancer research, if computational resources are plentiful but labeled training data are limited, large language models can be used for zero- or few-shot learning to achieve reasonable performance. When computational resources are more limited but labeled training data are readily available, even simple machine learning architectures can achieve good performance for such tasks.

**Supplementary Information:**

The online version contains supplementary material available at 10.1186/s12859-023-05439-1.

## Background

Precision oncology, defined as tailoring cancer treatment to the individual clinical and molecular characteristics of patients and their tumors [1], is an increasingly important goal in cancer medicine. This strategy requires linking tumor molecular data [2] to data on patient outcomes to ask research questions about the association between tumor characteristics and treatment effectiveness. Despite the increasing sophistication of molecular and bioinformatic techniques for genomic data collection, the ascertainment of corresponding clinical outcomes from patients who undergo molecular testing has remained a critical barrier to precision cancer research. Outside of therapeutic clinical trials, key clinical outcomes necessary to address major open questions in precision oncology, such as which biomarkers predict cancer response (improvement) and progression (worsening), are generally recorded only in the free text documents generated by radiologists and oncologists as they provide routine clinical care.

Clinical cancer outcomes other than overall survival are not generally captured in standard cancer registry workflows; historically, abstraction of such outcomes from the electronic health record (EHR) has therefore required resource-intensive manual annotation. If this abstraction has occurred at all, it has generally been performed within individual research groups in the absence of data standards, yielding datasets of questionable generalizability. To address this gap, our research group developed the ‘PRISSMM’ framework for EHR review. PRISSMM is a structured rubric for manual annotation of each pathology, radiology/imaging, and medical oncologist report to ascertain cancer features and outcomes; each imaging report is reviewed in its own right to determine whether it describes cancer response, progression, or neither [3]. This annotation process also effectively yields document labels that can be used to train machine learning-based natural language processing (NLP) models to recapitulate these manual annotations. We previously detailed the PRISSMM annotation directives for ascertaining cancer outcomes and demonstrated the feasibility of using PRISSMM labels to train NLP models that can identify cancer outcomes within imaging reports [3, 4] and medical oncologist notes [4, 5].

While applying NLP to clinical documents can dramatically accelerate outcome ascertainment, training these models from randomly initialized weights remains resource-intensive, requiring thousands of manually annotated documents. Modern advances in NLP could reduce this data labeling burden. Semi-supervised learning techniques based on language modeling, or using components of a sentence or document to predict the remainder of the text, have become cornerstones of NLP [6]. The Universal Language Model Fine-Tuning technique demonstrated that by first training a language model on a large general text corpus and then further pre-training it on in-domain text, it is possible to fine-tune useful text classifiers using far fewer labeled examples than might otherwise be required [7]. Simultaneously, the Transformer architecture [8] and many of its derivatives, such as Bidirectional Encoder Representations from Transformers (BERT) [9] and BERT versions fine-tuned on clinical text [10] have facilitated training of high-performing, large language models. These architectures have often been designed around processing relatively short segments of text, but methods for applying them to longer documents, including the Transformer-XL [11], Reformer [12], and Longformer [13] architectures, have also been developed. Transformer-based models have been applied to radiology reports and found to outperform simpler methods at certain general medical annotation tasks [14–17]. These models also facilitate the emerging paradigm of zero-shot Learning, in which a scaled-up, pretrained language model is primed for NLP tasks via conditional language generation. It has been successful in general domain NLP [18] for different tasks. Specifically for question-answering tasks, the Text-to-Text Transfer Transformer [19] with instruction finetuning [20] has yielded impressive results for reasonable model sizes. To our knowledge, preceding investigations have not yielded competitive performance on biomedical NLP tasks while utilizing general LLMs [21]. The practical utility of these architectures for ascertaining cancer outcomes in clinical research settings using limited quantities of labeled EHR text is unknown.

In this study, we evaluated the performance of various NLP architectures at capturing cancer response and progression from imaging reports for a cohort of patients with lung cancer. Candidate architectures included simple ‘bag of words’ linear models and convolutional neural networks [22], as well as Transformer architectures with language model pretraining. We varied the size of the training dataset for each architecture to evaluate the association of architecture with the quantity of labeled data required to train high-performing models.

## Results

**Fig. 1:**
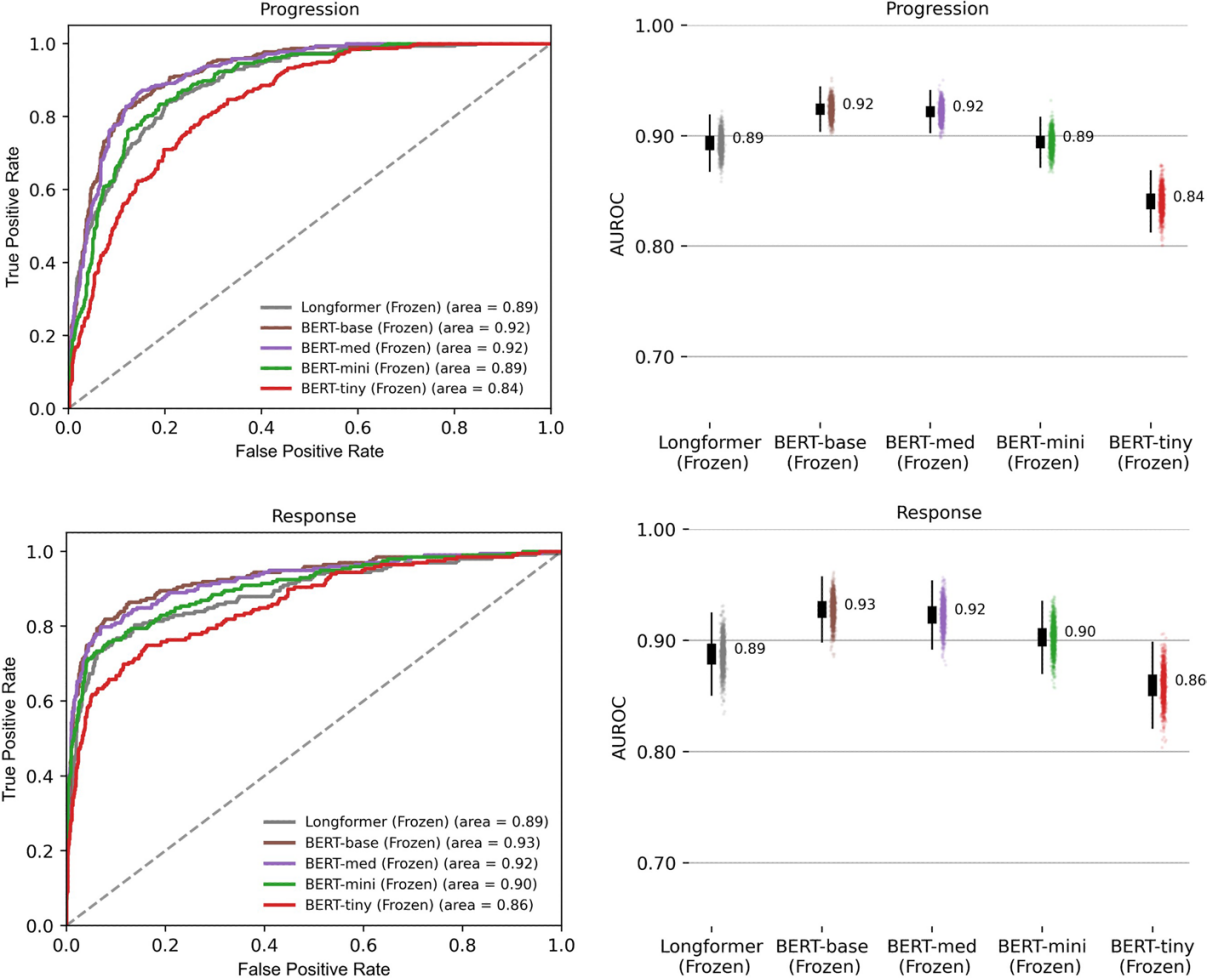
Impact of BERT model architecture on performance. Performance of Transformer-based architectures (with the language model frozen) for the document classification tasks of identifying cancer progression/worsening and response/improvement. In this figure, all architectures were fine-tuned directly on the classification tasks, using a convolutional neural network head, without language model pre-training. For boxplots in the right column, the middle line represents the median, the lower and upper hinges correspond to the 1st and 3rd quartiles, and the whisker corresponds to the minimum or maximum values no further than 1.5 times the inter-quartile range from the hinge. Data beyond the whiskers are outlying points, plotted individually in the scatter plots

We first examined the impact of model architecture, number of parameters, language model domain adaptation, and classification head structure on the performance of BERT-based models for ascertaining cancer response/improvement or progression/worsening from imaging reports. With no domain adaptation, a frozen language model, and a CNN classification head, the BERT-base model yielded AUROC of 0.93 for capturing response/improvement and 0.92 for cancer progression/worsening, outperforming BERT-tiny, BERT-mini, BERT-med, and a Longformer architecture (Fig. 1, Table 1). The metrics of AUPRC, accuracy, precision, recall, MCC, and F1 scores are provided in Table 1 and shown in Additional file 1: Figs. 1–2. Additional model characteristics are provided in Table 2.

**Table 1:** Performance of Transformer-based architectures compared to baseline models

	Progression						
	Accuracy	Precision	AUROC [95% CI]	F1	AUPRC	Recall	MCC
BERT-Base	0.88 [0.87, 0.90]	0.71 [0.66, 0.76]	0.92 [0.91, 0.94]	0.72 [0.68, 0.76]	0.76 [0.72, 0.81]	0.74 [0.69, 0.79]	0.65 [0.60, 0.70]
BERT-Med	0.88 [0.86, 0.89]	0.69 [0.64, 0.73]	0.92 [0.91, 0.94]	0.73 [0.69, 0.76]	0.75 [0.69, 0.80]	0.77 [0.72, 0.81]	0.65 [0.60, 0.69]
BERT-Mini	0.85 [0.83, 0.87]	0.61 [0.56, 0.66]	0.89 [0.88, 0.91]	0.68 [0.64, 0.72]	0.68 [0.62, 0.73]	0.77 [0.72, 0.81]	0.59 [0.54, 0.63]
BERT-Tiny	0.80 [0.78, 0.82]	0.51 [0.46, 0.56]	0.84 [0.82, 0.86]	0.56 [0.52, 0.61]	0.56 [0.50, 0.63]	0.63 [0.58, 0.68]	0.43 [0.38, 0.49]
Longformer	0.86 [0.84, 0.87]	0.70 [0.63, 0.75]	0.89 [0.87, 0.91]	0.62 [0.57, 0.67]	0.70 [0.65, 0.75]	0.55 [0.50, 0.61]	0.54 [0.48, 0.59]
Clinical BERT	0.88 [0.86, 0.89]	0.69 [0.64, 0.74]	0.93 [0.91, 0.94]	0.72 [0.68, 0.75]	0.77 [0.72, 0.82]	0.75 [0.70, 0.80]	0.64 [0.59, 0.69]
DFCI-ImagingBERT (BERT frozen, CNN head)	0.90 [0.89, 0.92]	0.75 [0.70, 0.79]	**0.95** [0.94, 0.96]	0.78 [0.74, 0.81]	0.84 [0.80, 0.87]	0.81 [0.77, 0.85]	0.72 [0.68, 0.76]
DFCI-ImagingBERT (BERT unfrozen, linear head)	0.90 [0.89, 0.92]	0.74 [0.69, 0.79]	**0.95** [0.94, 0.96]	0.78 [0.74, 0.81]	0.85 [0.81, 0.89]	0.81 [0.77, 0.85]	0.71 [0.67, 0.76]
CNN	0.89 [0.87, 0.90]	0.72 [0.66, 0.76]	0.93 [0.92, 0.95]	0.74 [0.70, 0.78]	0.81 [0.77, 0.85]	0.77 [0.72, 0.82]	0.67 [0.62, 0.72]
TF-IDF	0.88 [0.86, 0.89]	0.72 [0.67, 0.77]	0.92 [0.90, 0.93]	0.69 [0.64, 0.73]	0.75 [0.71, 0.80]	0.66 [0.61, 0.71]	0.61 [0.56, 0.66]
Flan-T5-XXL (zero-shot)	0.89 [0.87, 0.90]	0.77 [0.72, 0.82]	0.92 [0.91, 0.94]	0.71 [0.66, 0.75]	0.77 [0.72, 0.81]	0.65 [0.60, 0.71]	0.64 [0.59, 0.69]

	Response						
	Accuracy	Precision	AUROC [95% CI]	F1	AUPRC	Recall	MCC
BERT-Base	0.93 [0.92, 0.95]	0.80 [0.74, 0.85]	0.93 [0.90, 0.95]	0.73 [0.68, 0.78]	0.78 [0.73, 0.83]	0.67 [0.61, 0.74]	0.70 [0.64, 0.75]
BERT-Med	0.93 [0.92, 0.94]	0.75 [0.69, 0.81]	0.92 [0.90, 0.95]	0.71 [0.66, 0.76]	0.78 [0.72, 0.83]	0.68 [0.62, 0.74]	0.67 [0.62, 0.73]
BERT-Mini	0.92 [0.91, 0.94]	0.72 [0.65, 0.78]	0.90 [0.88, 0.93]	0.71 [0.66, 0.76]	0.74 [0.67, 0.79]	0.71 [0.65, 0.77]	0.67 [0.61, 0.72]
BERT-Tiny	0.89 [0.88, 0.91]	0.59 [0.53, 0.66]	0.86 [0.83, 0.89]	0.61 [0.55, 0.67]	0.63 [0.57, 0.70]	0.63 [0.57, 0.70]	0.55 [0.49, 0.61]
Longformer	0.92 [0.90, 0.93]	0.80 [0.72, 0.87]	0.89 [0.86, 0.91]	0.61 [0.54, 0.67]	0.71 [0.64, 0.77]	0.49 [0.42, 0.56]	0.59 [0.52, 0.65]
Clinical BERT	0.93 [0.92, 0.94]	0.77 [0.70, 0.83]	0.93 [0.90, 0.95]	0.72 [0.66, 0.77]	0.77 [0.70, 0.83]	0.67 [0.61, 0.74]	0.68 [0.62, 0.73]
DFCI-ImagingBERT (BERT frozen, CNN head)	0.94 [0.93, 0.95]	0.83 [0.77, 0.89]	**0.94** [0.93, 0.96]	0.76 [0.71, 0.80]	0.81 [0.76, 0.86]	0.69 [0.63, 0.76]	0.73 [0.67, 0.78]
DFCI-ImagingBERT (BERT unfrozen, linear head)	0.94 [0.93, 0.95]	0.84 [0.77, 0.89]	**0.93** [0.91, 0.95]	0.73 [0.68, 0.78]	0.80 [0.75, 0.85]	0.65 [0.59, 0.72]	0.71 [0.65, 0.76]
CNN	0.93 [0.92, 0.94]	0.92 [0.86, 0.97]	**0.94** [0.92, 0.96]	0.67 [0.60, 0.72]	0.82 [0.77, 0.87]	0.52 [0.45, 0.59]	0.66 [0.60, 0.72]
TF-IDF	0.93 [0.91, 0.94]	0.81 [0.74, 0.87]	**0.93** [0.91, 0.95]	0.68 [0.63, 0.73]	0.75 [0.69, 0.81]	0.59 [0.53, 0.66]	0.65 [0.59, 0.71]
Flan-T5-XXL (zero-shot)	0.92 [0.90, 0.93]	0.69 [0.63, 0.76]	0.90 [0.87, 0.93]	0.69 [0.64, 0.74]	0.69 [0.61, 0.75]	0.68 [0.61, 0.75]	0.64 [0.58, 0.70]

Performance of Transformer-based architectures compared to baseline models for the document classification tasks of identifying cancer progression/worsening and response/improvement. Additional model characteristics are provided in Table 2. Precision, Recall, and F1 measures are calculated using the model output score threshold that maximizes the F1 score in the training set. The best AUROC for each outcome is in bold face, as are the AUROC’s for any model that are not statistically significantly different from the best AUROC for each outcome

**Fig. 2:**
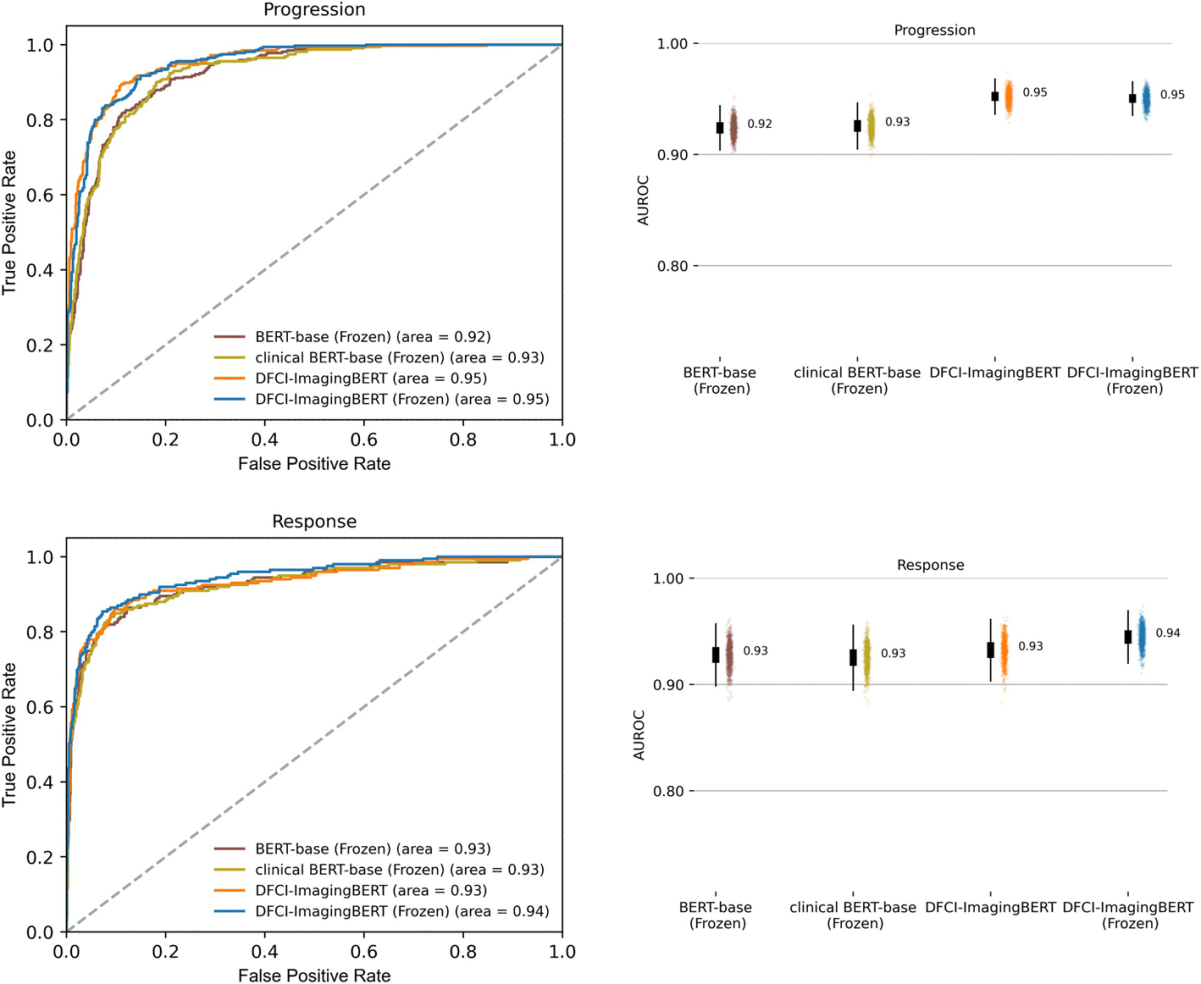
Impact of BERT model language model tuning on performance. Association between language model pre-training and ultimate classification model performance. BERT-base represents a BERT model without language model pre-training on clinical text; clinical BERT-base represents a BERT-base model, fine-tuned on intensive care unit EHR data; DFCI-ImagingBERT represents a BERT-base model, with its language model further pre-trained on in-domain imaging reports from our institution. Figure depicts results with language models that were frozen for downstream classification task. For boxplots in the right column, the middle line represents the median, the lower and upper hinges correspond to the 1st and 3rd quartiles, and the whisker corresponds to the minimum or maximum values no further than 1.5 times the inter-quartile range from the hinge. Data beyond the whiskers are outlying points, plotted individually in the scatter plots

After domain adaptation on imaging reports from our institution (N = 662,579 reports from 27,483 patients with multiple types of cancer) was performed beginning with a BERT-base model, the resulting language model (DFCI-ImagingBERT) was frozen and fine-tuned with a CNN classification head, yielding better performance than BERT-base, with AUROCs of 0.94 for ascertaining response/improvement and 0.95 for progression/worsening. Models based on ClinicalBERT yielded slightly lower performance compared to DFCI-ImagingBERT, with an AUROC of 0.93for both response and progression outcomes (Fig. 2, Table 1). AUPRC, accuracy, precision, recall, F1, and MCC scores for these models are shown in Additional file 1: Figs. 3–4.

**Fig. 3:**
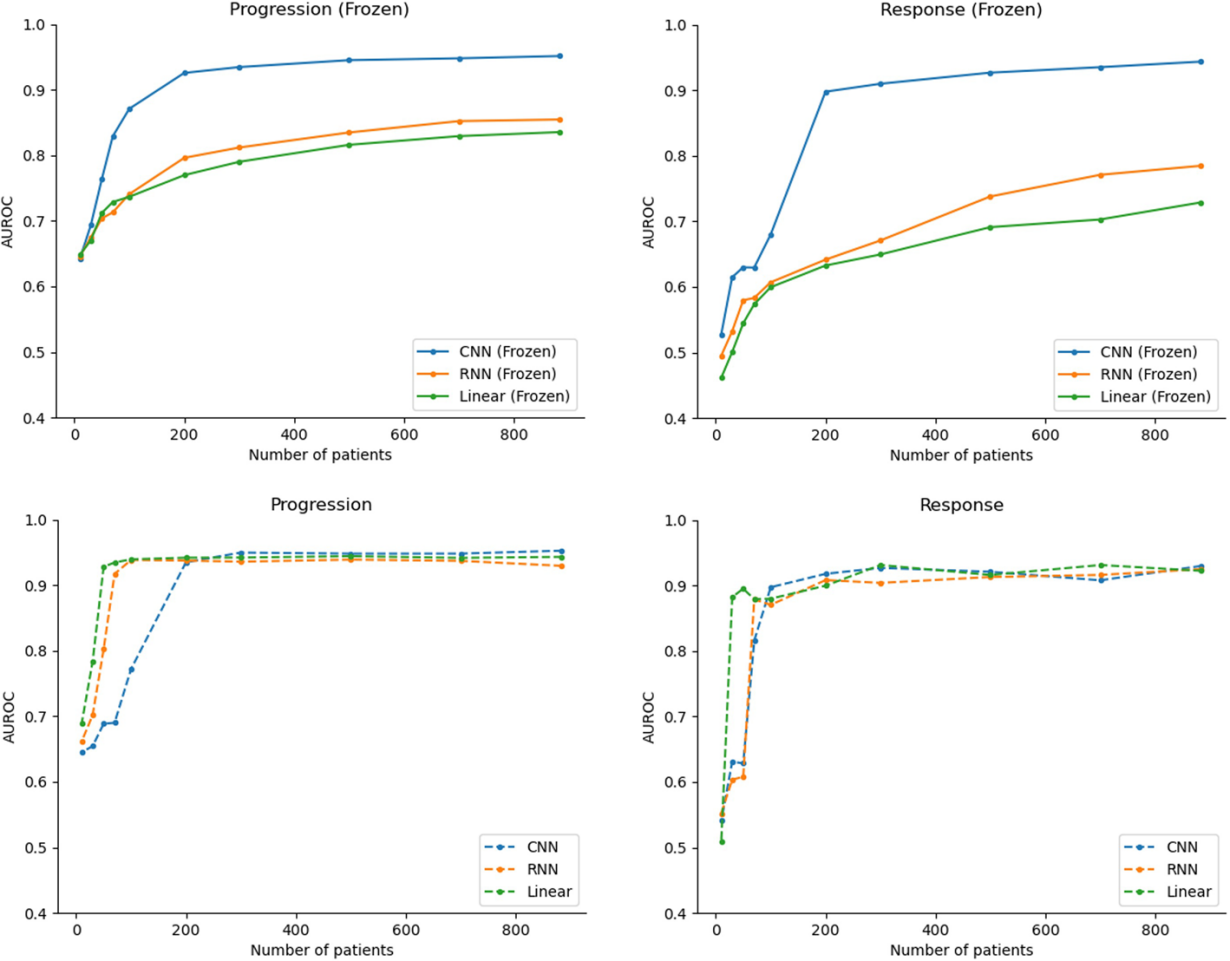
Impact of classification head on performance (DFCI-ImagingBERT). Associations among classification head, training dataset size, and model performance for progression/worsening (left) and response/improvement (right) for the DFCI-ImagingBERT architecture. CNN = convolutional neural network; RNN = recurrent neural network. Linear = fully connected layer applied to the BERT [CLS] token for each document

We also evaluated the impact of varying classification head architectures for DFCI-ImagingBERT, including a CNN; a linear layer based on the classification token vector; and an RNN (bidirectional gated recurrent unit) [23] architecture on model performance as a function of training set size (Fig. 3, Additional file 1: Fig. 5). When the language model layers were frozen, the CNN head was associated with the best performance at the largest training set size, yielding the performance metrics described above. The RNN head yielded AUROCs of 0.78 for response/improvement and 0.85 for progression/worsening; the linear head yielded AUROCs of 0.72 for response/improvement and 0.84 for progression/worsening. With the language model unfrozen, given the full training set, the linear head yielded AUROCs of 0.92 and 0.94, and the CNN was best with AUROCs of 0.93 and 0.95 for response and progression outcomes respectively. The linear head, however, yielded better performance at smaller training set sizes (Fig. 3).

**Fig. 4:**
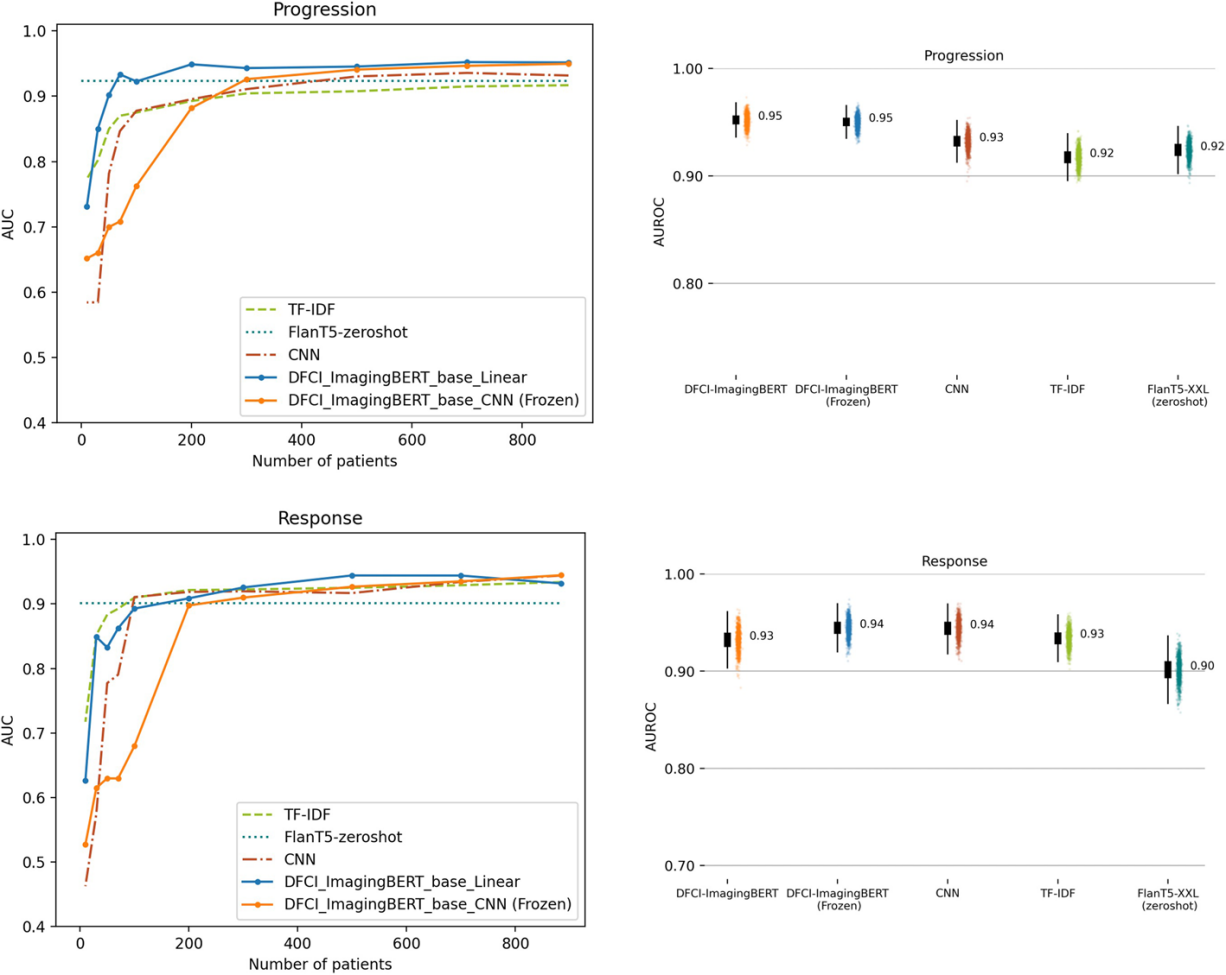
Comparing DFCI-ImagingBERT model performance to baseline models. Model performance as a function of architecture and training dataset size for identifying progression/worsening (top row) and response/improvement (bottom row). For boxplots in the right column, the middle line represents the median, the lower and upper hinges correspond to the 1st and 3rd quartiles, and the whisker corresponds to the minimum or maximum values no further than 1.5 times the inter-quartile range from the hinge. Data beyond the whiskers are outlying points, plotted individually in the scatter plots. TF-IDF: term frequency-inverse document frequency. CNN: convolutional neural network

We therefore selected DFCI-ImagingBERT as the BERT-based model to compare against the simple term frequency-inverse document frequency (TF-IDF) and CNN neural network architectures. The performance of each of these architectures as a function of training set size is depicted in Fig. 4. For the response/improvement outcome, DFCI-ImagingBERT with a frozen language model and CNN classification head yielded the best performance, with an AUROC of 0.94 after training on the full training dataset; DFCI-ImagingBERT with an unfrozen language model and linear head yielded an AUROC of 0.93; the simple CNN model yielded an AUROC of 0.94; and the TF-IDF model yielded an AUROC of 0.93. For the progression/worsening outcome, DFCI-ImagingBERT (either with a frozen language model and CNN head or an unfrozen language model and linear head) yielded the best performance, with an AUROC of 0.95; followed by the CNN, with an AUROC of 0.93; and the TF-IDF model, with an AUROC of 0.92. The largest gains in model performance were achieved when the training set size increased from reports for 50 patients up to reports for 300 patients, with diminishing returns as training set size increased further thereafter. For the progression outcome, DFCI-ImagingBERT with an unfrozen language model and linear classification head performed best on samples of patients smaller than 300; for the response outcome, the TF-IDF model performed best on such samples (Fig. 4).

Finally, we evaluated the performance of the Flan-T5-XXL text to text model for zero-shot learning for these tasks, with no weight updates and limited prompt engineering and hyperparameter tuning. This model achieved AUROC of 0.92 for the progression/worsening task and AUROC of 0.90 for the response/improvement task.

## Discussion

Natural language processing has the potential to substantially accelerate precision oncology research by enabling observational clinical outcomes to be linked to molecular cancer data for downstream analysis, increasing the sample size of clinico-genomic datasets several times over [4]. This process allows outcomes to be ascertained on a timepoint-specific basis, facilitating analysis for research questions that may focus on different portions of the disease trajectory. NLP could also inform cancer care delivery by processing EHR data to identify patients who have specific disease states at individual moments in time, and who could therefore benefit from interventions such as clinical trial enrollment or palliative care services.

Transformer-based models have become standard for general NLP tasks given their potential to yield improved performance, potentially while relying upon less labeled data to train supervised learning models. We found that a BERT model with domain adaptation on text from our institution performed better than simpler TF-IDF and CNN models for text classification, but the simple models still yielded AUROCs > 0.9, such that depending on end use case and computational resources, complex models may not always be needed if training data are readily available. On the other hand, we found that the Flan-T5-XXL architecture with a small amount of prompt engineering yielded good zero-shot performance with no domain adaptation pretraining or fine-tuning on labeled data, demonstrating the potential utility of large language models in this space when computational resources are readily available.

There are several potential explanations for the similar performance observed between a Transformer architecture and simpler models for this clinical text classification task, particularly for the response/improvement outcome. One possibility is that the outcomes are distinctly keyword-sensitive, such that a few words within long documents may define outcomes with relatively less dependence on other context from the document. Clinical imaging reports are also substantially longer than typical sequence lengths for standard Transformer models; this may dilute the benefits derived from contextual token embedding by language models in other contexts. Still, we also evaluated the Longformer, which is a Transformer specifically designed for longer documents, and we did not observe improved performance for downstream classification tasks compared with a BERT architecture.

Strengths of this analysis include its derivation from a previously described labeled dataset of cancer outcomes linked to imaging reports; labels in this dataset have been shown to be clinically relevant and associated with overall survival [4]. Our results provide practical guidance to researchers who may seek to gather just the necessary volume of labeled clinical data in order to train NLP models to perform cancer outcome extraction. We found that, as expected, model performance improves with greater training set size, but that the marginal improvement once the training set reached 300 patients (~ 3000 imaging reports) was relatively small.

Limitations include the single-institution nature of the data, as well as limited hyperparameter tuning. Transformers are notoriously challenging to train, requiring specialized learning rate schedules and initialization strategies for optimization [24]. Given the computational complexity of domain adaptation and classification fine-tuning for Transformer models, it was not feasible to perform automated (e.g., grid search) hyperparameter tuning for this analysis. It is possible that any of our models might have performed better with additional tuning, and that Transformers might yield more improvements on different EHR data tasks. Many variants of healthcare-relevant language models exist [10, 25]; we chose one trained on electronic health records text, rather than academic publications, as a basis for evaluation. This precludes sharing the DFCI-ImagingBERT model weights beyond our institution to evaluate external generalizability. However, our goal in this analysis is to provide practical guidance for small teams seeking to extract cancer outcomes at academic medical centers, which may have computational resources comparable to those in our study, not to train models for external application. NLP models trained on institutional protected health information may carry at least some risk of exposing that information to adversarial attacks [26], and further research into best practices for generalizable cross-institution NLP healthcare modeling is needed.

## Conclusion

We conducted a systematic evaluation of NLP models for extracting clinical cancer outcomes from EHR data. A BERT model with domain adaptation and supervised fine-tuning for classification yielded the best performance across tasks and metrics, though simpler models demonstrated good performance given large quantities of training data. Zero-shot learning based on modern large language models also demonstrated good performance on some metrics. The reported quantitative results suggest that when developing AI models to extract outcomes from imaging reports for clinical cancer research, if computational resources are plentiful but labeled training data are limited, large language models can be used for zero- or few-shot learning to achieve reasonable performance. When computational resources are more limited but labeled training data are readily available, even simple machine learning architectures can achieve good performance for such tasks.

## Methods

### Cohort

The overall cohort for this analysis consisted of patients with cancer participating in a single-institution genomic profiling study [27], and relevant data consisted of imaging reports for each patient. Each report was treated as its own unit of analysis, and reports were divided, at the patient level, into training (80%), validation (10%), and test (10%) datasets.

For language model pre-training on data from our institution, reports for all patients in the training set were included. This dataset included 662,579 reports from 27,483 patients with multiple types of cancer whose tumors were sequenced through our institutional precision medicine study [27].

For classification model training, the imaging reports for a subset of patients with lung cancer were manually annotated to ascertain the presence of cancer response or progression in each report using the PRISSMM framework, as previously described [3]. Briefly, during manual annotation, human reviewers recorded whether each imaging report indicated any cancer, and if so, whether it was responding/improving, progressing/worsening, stable (neither improving nor worsening), mixed (with some areas improving and some worsening), or indeterminate (if assigning a category was not possible due to radiologist uncertainty or other factors). For NLP model training, response/improvement and progression/worsening were each treated as binary outcomes, such that an imaging report indicating no cancer, or indicating stable, mixed, or indeterminate cancer status, was coded as neither improving nor worsening. This process, and interrater reliability statistics for manual annotation, have been described previously [3]. The classification dataset consisted of 14,218 labeled imaging reports for 1112 patients. Among the reports, 1635 (11.5%) indicated cancer response/improvement, and 3522 (24.8%) indicated cancer progression/worsening.

### Models

Our baseline architecture was a simple logistic regression model in which the text of each imaging report was vectorized using term frequency-inverse document frequency (TF-IDF) vectorization [28]. This model used elastic net regularization with alpha = 0.0001, L1 ratio of 0.15, and was trained with stochastic gradient descent. Other architectures included one-dimensional convolutional neural networks (CNNs) [22] and Transformer-based [8] networks. For the CNNs, text was tokenized and numericalized using the Tensorflow Keras tokenizer, with a vocabulary size of 20,000. For Transformer networks, the Huggingface tokenizer [29] corresponding to each Transformer architecture was applied. We first evaluated a convolutional neural network architecture (CNN), trained only using our labeled data, as previously described [3], except with only one output per model. Next, we evaluated classification heads based on BERT models trained on general domain text only, using progressively larger numbers of parameters (BERT-tiny, BERT-mini, BERT-med, or BERT-base); as well as a Longformer model. We next evaluated BERT models adapted on general medical text (ClinicalBERT) [10], or first adapted from general domain BERT-base on in-domain imaging reports from our institution (DFCI-ImagingBERT). For BERT models, text was truncated and padded to the maximum sequence length of the model (512 tokens) beginning from the end of the document. For the CNN model, text was truncated and padded to a length of 1000 tokens beginning from the end of the document. For the Longformer, text was truncated and padded to a length of 1024 tokens from the end of the document. Training and evaluation were performed using Pytorch [30] and Tensorflow [31].

Subsequently, we conducted an evaluation of zero-shot learning using the T5 encoder-decoder model [19] based on the Transformer architecture, and the Flan-T5 model [20], which is an instruction-finetuned variant of T5 that has demonstrated good performance across various natural language processing tasks. We additionally evaluated OPT [32] models up to 30B size, T0 [33] models, and some models pretrained on clinical/medical domain corpora, namely ClinicalT5 [34], Clinical-T5 [35], and SciFive [36]. All of these models yielded only very modest results on our validation cohort, compared to Flan-T5 in XXL size, which we chose for our further analysis. We employed T5ForConditionalGeneration and corresponding tokenizer from the Huggingface transformers library [29] with the following input text template: "question: {question} context: {imaging report}”. For the response/improvement task, the corresponding question text was "Is there improvement/response/shrinking of cancer (yes/no)?" Similarly, for the progression/worsening task, the question text was "Is there worsening of cancer (yes/no)?." The selection of questions for each task involved a limited amount of manual prompt engineering identifying the questions that exhibited the best performance on the validation set. The T5 model’s utilization of relative positional embeddings enabled us to use full input texts without truncation. To determine the classification output, we adopted a specific approach where we allowed the model to generate an output text and extracted the first token logits of the generated text. The probability of the "yes" class was computed using softmax(1 − logit of the "no" token). This method demonstrated superior performance on the validation set compared with using the logit of the "yes" class directly.

### Model training

For BERT model domain adaptation on imaging reports from our institution (DFCI-ImagingBERT), the base model was BERT-base, pre-trained on general domain text and accessed using the Huggingface library. Pre-training was performed over 10 epochs, which took 10.5 days on a single machine equipped with an NVIDIA Tesla T4 GPU (16 GB GDDR6).

For classification models, separate binary prediction models were trained to identify response/improvement and progression/worsening, since these constitute distinct outcomes that would be used differently for downstream analyses (e.g., calculating response rate in a given time period, versus progression-free survival). A binary cross-entropy loss function and the Adam optimizer were applied for training for the TF-IDF and CNN models, and the AdamW (Adam with weight decay) optimizer [37] was applied for BERT and Longformer-based models.

We trained each model using fixed samples of reports from the training set, corresponding to the reports from 10, 30, 50, 70, 100, 200, 300, 500, 700, or 884 patients, to evaluate the rate at which performance of each architecture improved using progressively more training examples. For BERT-based models, experiments were also conducted to examine the effect of various classification head architectures, including linear, convolutional, and recurrent neural network architectures, on model performance; and to evaluate the impact of freezing the weights of the underlying language model when fine-tuning for classification. The full text of radiology reports—that is, the findings concatenated to the impression—was used for each model. BERT-based models have a sequence length limitation of 512 tokens, so for these models, the final 512 tokens of each report were used. For the Longformer model, the final 1024 tokens were used. For the simple CNN model, a sequence length of 1000 tokens was used. Reports shorter than the maximum length were padded to the maximum length. The time needed to train a DFCI-ImagingBERT model for a classification task on the full training set was 2.8 h on a machine equipped with a single NVIDIA T4 GPU. The time needed to train a Longformer model for a classification task on the full training set was 5.7 h on the same machine. To reflect a real-world scenario in which a small team of academic researchers seeks to extract cancer outcomes from EHR data with relatively limited computational resources, limited hyperparameter tuning was performed. For the TF-IDF model, different regularization approaches (L1, L2, and elastic net regularization) were tried. Hyperparameters for each model were tuned based on evaluation in the validation set. Model training code is provided at https://github.com/marakeby/clinicalNLP2.

### Model evaluation

Classification performance for outcomes (1) cancer response and (2) cancer progression was evaluated using the area under the receiver operating characteristic curve (AUROC) and the area under the precision-recall curve (AUPRC). Additional metrics including accuracy, precision, recall, Matthew correlation coefficient (MCC) [38] and F1 score are presented; for all fine-tuned models, the threshold model output for a positive prediction was defined as the best F1 threshold in the training set; for Flan-T5 zero-shot, the threshold probability was set to 0.5. After training was complete, models were evaluated using data for held-out test set patients, after which no further training was performed. Figures were then generated to illustrate the performance of each architecture given specific training set sizes. Interquartile ranges for model performance metrics were calculated using a bootstrapping approach (evaluation on repeated random subsets of the test set). The AUROC of each model was compared to the AUROC of the best model statistically using two-sided alpha of 0.05 based on the bootstrapping; no adjustment for multiplicity was performed.

**Table 2:** Model characteristics

Model	Architecture	# of parameters Trainable/Total	Pre-trained or contextual token embeddings?	Language model pre-trained in domain?	Language model frozen for classification training?	Final classification training layer/strategy tested
TF-IDF	Bag of words logistic regression with elastic net regularization	40 K	No	N/A	N/A	N/A
CNN	One-dimensional convolutional neural network with global max-pooling	7 M	No	N/A	N/A	N/A
BERT-base	BERT	766 K/110 M	Yes	No	Yes	CNN head
BERT-med	BERT	521 K/42 M	Yes	No	Yes	CNN head
BERT-mini	BERT	275 K/11 M	Yes	No	Yes	CNN head
BERT-tiny	BERT	152 K/4.5 M	Yes	No	Yes	CNN head
Longformer [13]	RoBERTa with local context and global attention	766 K/128 M	Yes	No	Yes	CNN head
ClinicalBERT [10]	BERT	766 K/110 M	Yes	Partial (trained on MIMIC-III ICU data) [39]	Yes	CNN head
DFCI-ImagingBERT, frozen	BERT	766 K/110 M	Yes	Yes (trained on DFCI imaging reports)	Yes	CNN head
DFCI-ImagingBERT, unfrozen	BERT	110 M	Yes	Yes (trained on DFCI imaging reports)	No	Linear head
Flan-T5 XXL	Text to Text Transfer Transformer	11 B	Yes	No	N/A (zero-shot learning only)	1−the predicted probability of the word “no”

*TF-IDF* Term Frequency-Inverse Document Frequency, *CNN* convolutional neural network, *BERT* Bidirectional Encoder Representations from Transformers [9], *RoBERTa*, Robustly optimized BERT approach [40], *MIMIC* Medical Information Mart for Intensive Care, *DFCI* Dana-Farber Cancer Institute

### Supplementary Information

**Additional file 1:** Supplementary Figures.

## Data Availability

The underlying EHR text reports used to train and evaluate NLP models for these analyses constitute protected health information for DFCI patients and therefore cannot be made publicly available. Researchers with DFCI appointments and Institutional Review Board (IRB) approval can access the data on request. For external researchers, access would require collaboration with the authors and eligibility for a DFCI appointment per DFCI policies. Scripts used to implement, train, and evaluate the models are deposited in the public repository https://github.com/marakeby/clinicalNLP2.
