# Supplementary material for: Empirical evaluation of language modeling to ascertain cancer outcomes from clinical text reports
Source: BMC Bioinformatics. 2023 Sep 2;24:328. doi: 10.1186/s12859-023-05439-1 (PMC10474750; doi:10.1186/s12859-023-05439-1)

Supplementary Materials

Supplementary Figure 1: Performance of Transformer-based architectures for the document classification tasks of identifying response/improvement. All architectures were fine-tuned directly on the classification tasks, using a convolutional neural network head, without language model fine-tuning. a) Precision-Recall curve with the corresponding area under the curve shown in the legend. Different performance metrics are shown in the boxplots; area under precision recall curve (AUPRC) (b), accuracy (c), recall (d), precision (e), F1 (f) and MCC (g). Confusion matrices are shown for Longformer (h), BERT-base (i), BERT-med (j), BERT-mini (k), and BERT-tiny (l).

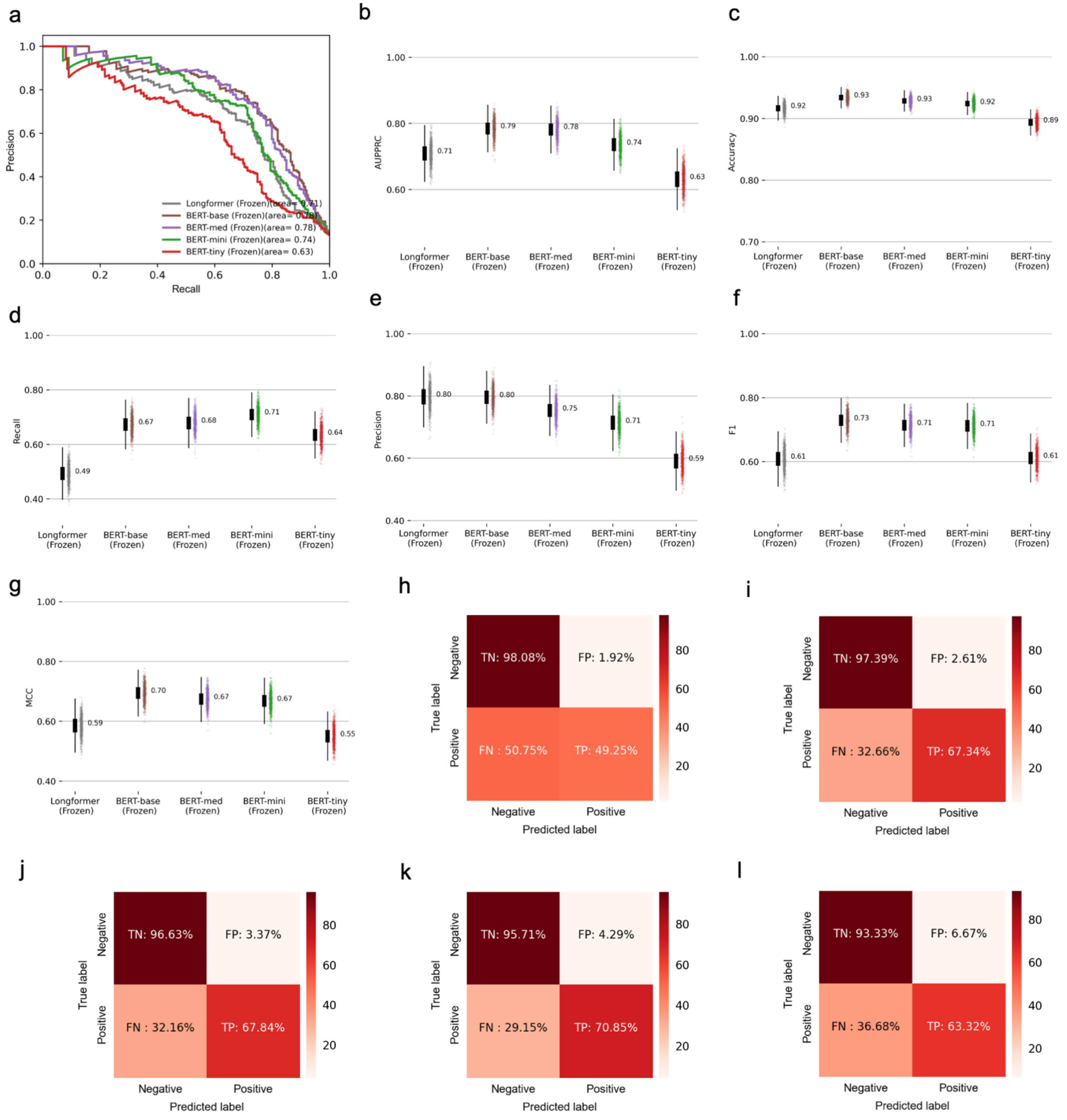

Supplementary Figure 2: Performance of Transformer-based architectures for the document classification tasks of identifying cancer progression/worsening. All architectures were fine-tuned directly on the classification tasks, using a convolutional neural network head, without language model fine-tuning. a) Precision-Recall curve with the corresponding area under the curve shown in the legend. Different performance metrics are shown in the boxplots; area under precision recall curve (AUPRC) (b), accuracy (c), recall (d), precision (e), F1 (f) and MCC (g). Confusion matrices are shown for Longformer (h), BERT-base (i), BERT-med (j), BERT-mini (k), and BERT-tiny (l).

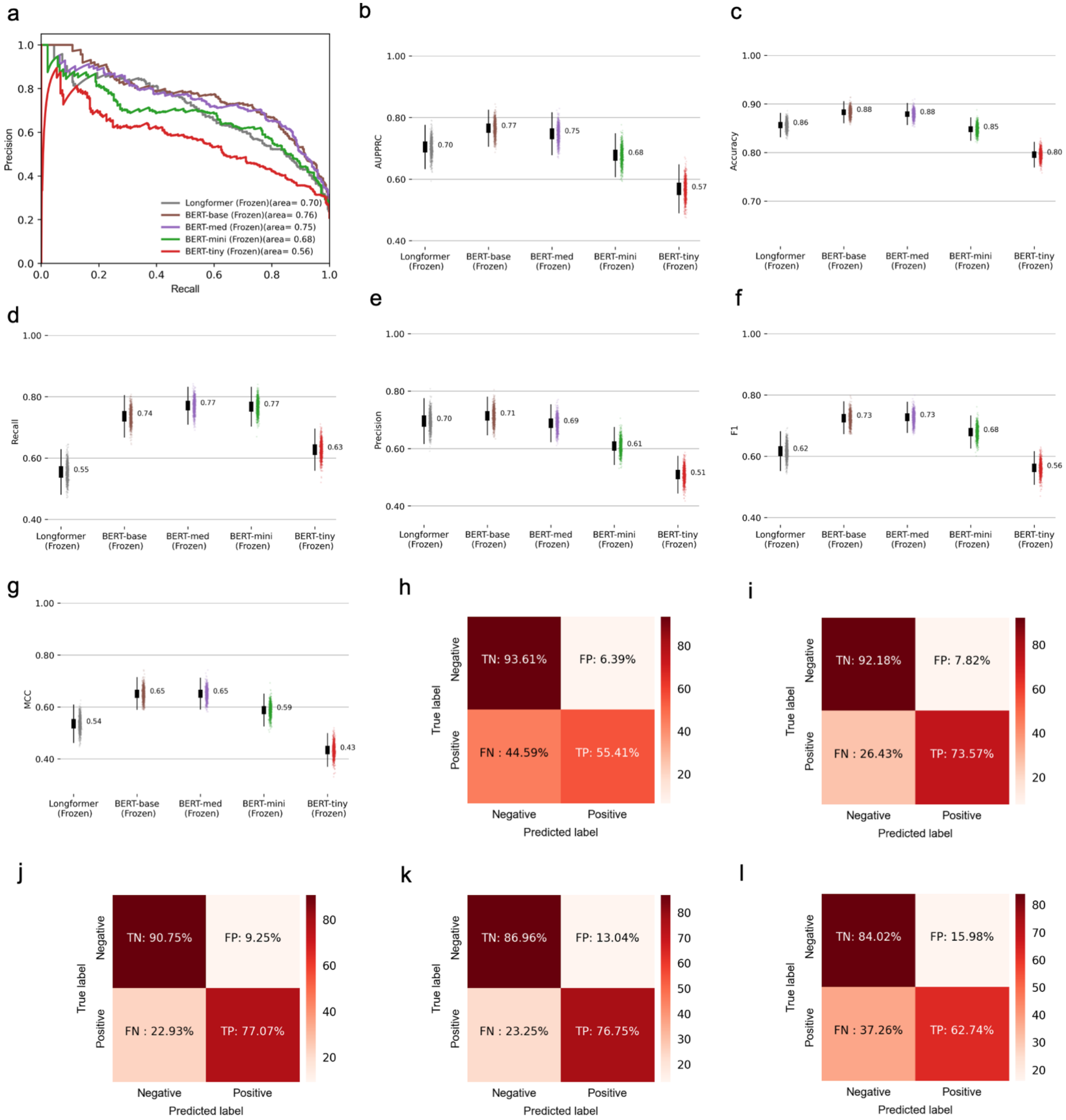

Supplementary Figure 3: The effect of language model fine-tuning on the response prediction performance. BERT-base represents a BERT model without language model fine-tuning on clinical text; clinical BERT-base represents a BERT-base model, fine-tuned on intensive care unit EHR data; DFCI-ImagingBERT represents a BERT-base model, with its language model fine-tuned on in-domain imaging reports from our institution. a) Precision-Recall curve with the corresponding area under the curve shown in the legend. Different performance metrics are shown in the boxplots; area under precision recall curve (AUPRC) (b), accuracy (c), recall (d), precision (e), and F1 (f). Confusion matrices are shown for BERT-base (g), clinical BERT-base (h), and DFCI-ImagingBERT(i).

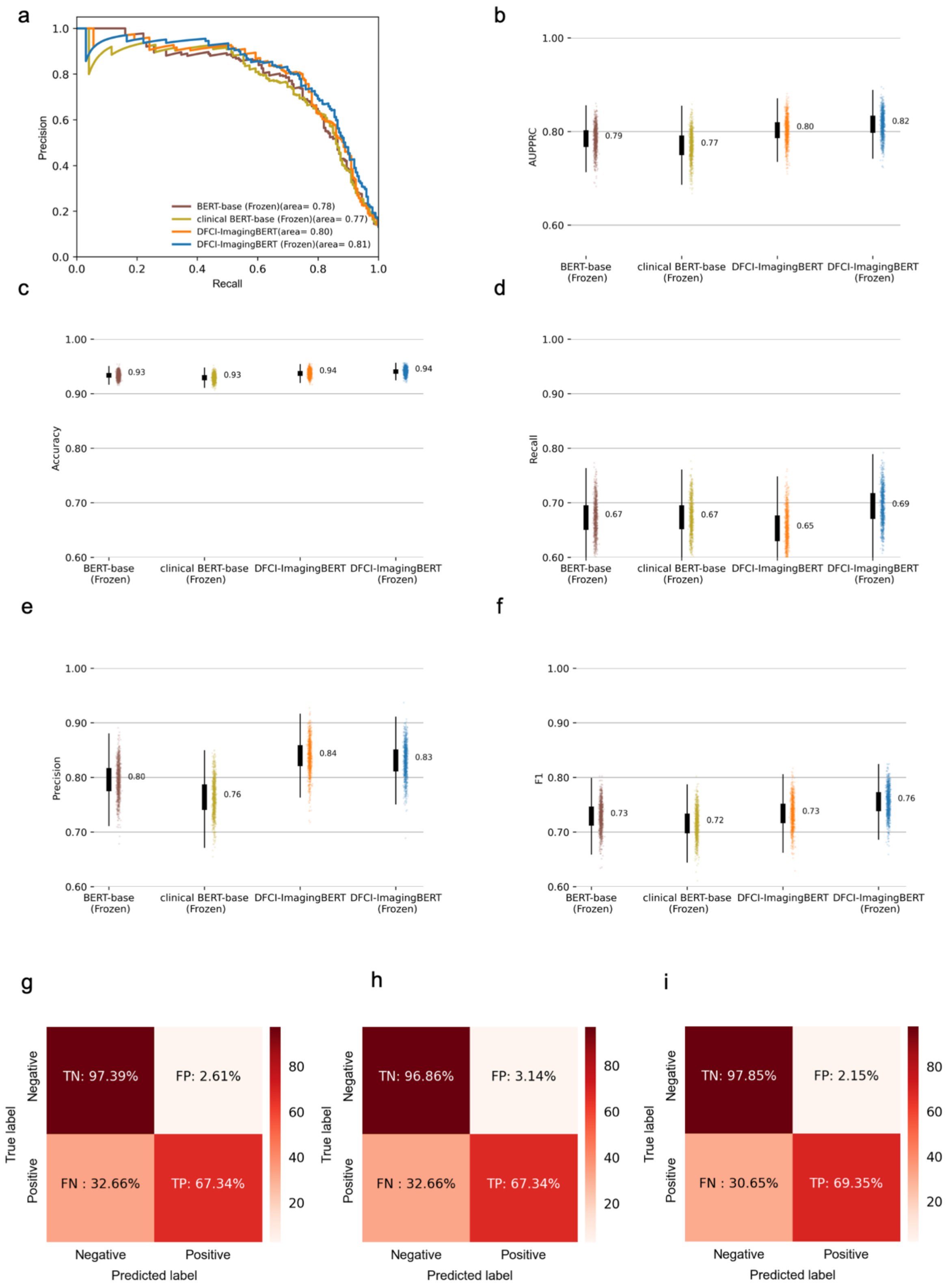

Supplementary Figure 4: The effect of language model fine-tuning on the cancer progression/worsening prediction performance. BERT-base represents a BERT model without language model fine-tuning on clinical text; clinical BERT-base represents a BERT-base model, fine-tuned on intensive care unit EHR data; DFCI-ImagingBERT represents a BERT-base model, with its language model fine-tuned on in-domain imaging reports from our institution. a) Precision-Recall curve with the corresponding area under the curve shown in the legend. Different performance metrics are shown in the boxplots; area under precision recall curve (AUPRC) (b), accuracy (c), recall (d), precision (e), and F1 (f). Confusion matrices are shown for BERT-base (g), clinical BERT-base (h), and DFCI-ImagingBERT(i).

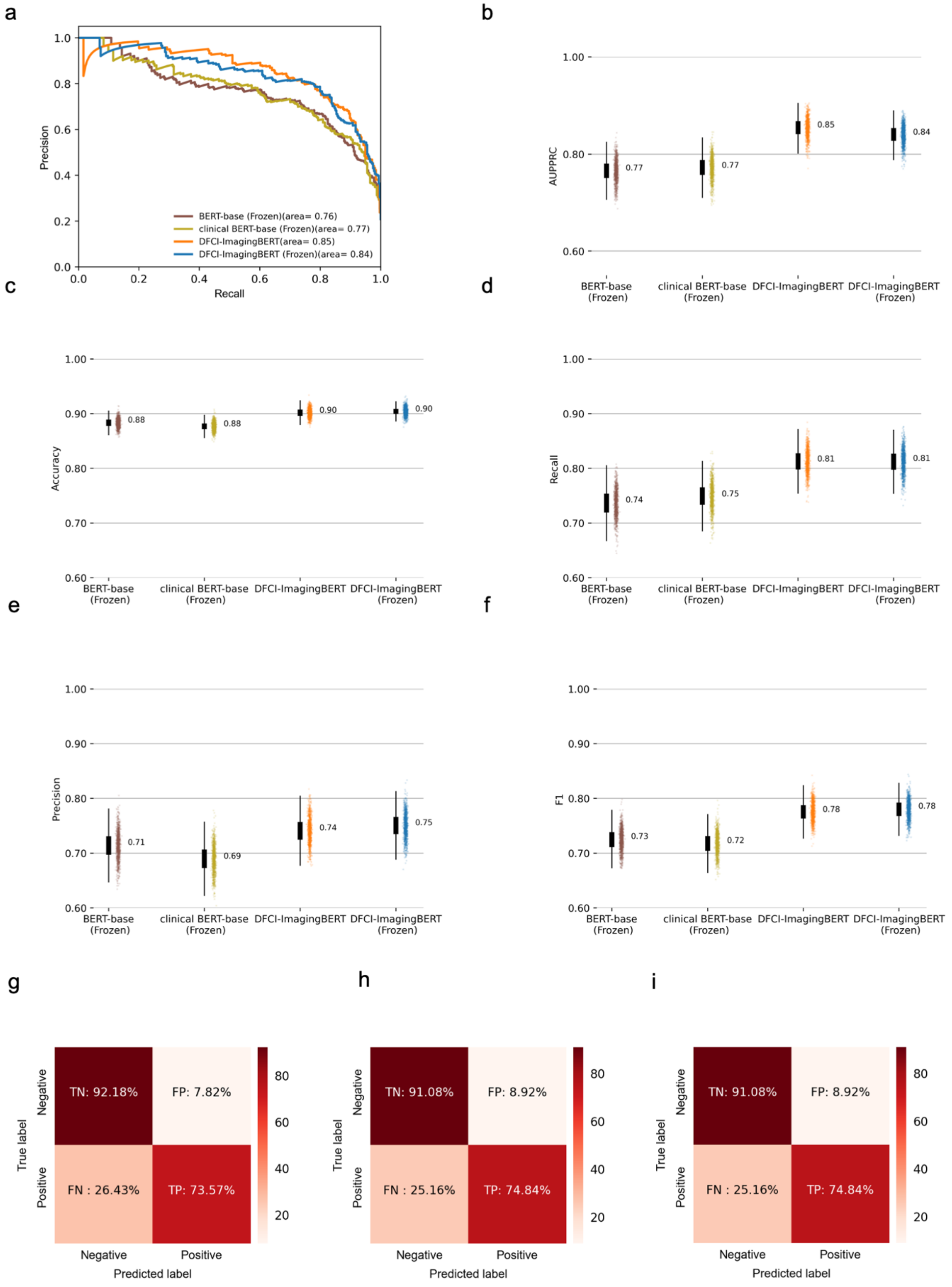

Supplementary Figure 5: Model performance as a function of architecture and training dataset size for identifying progression/worsening (top row) and response/improvement (bottom row).

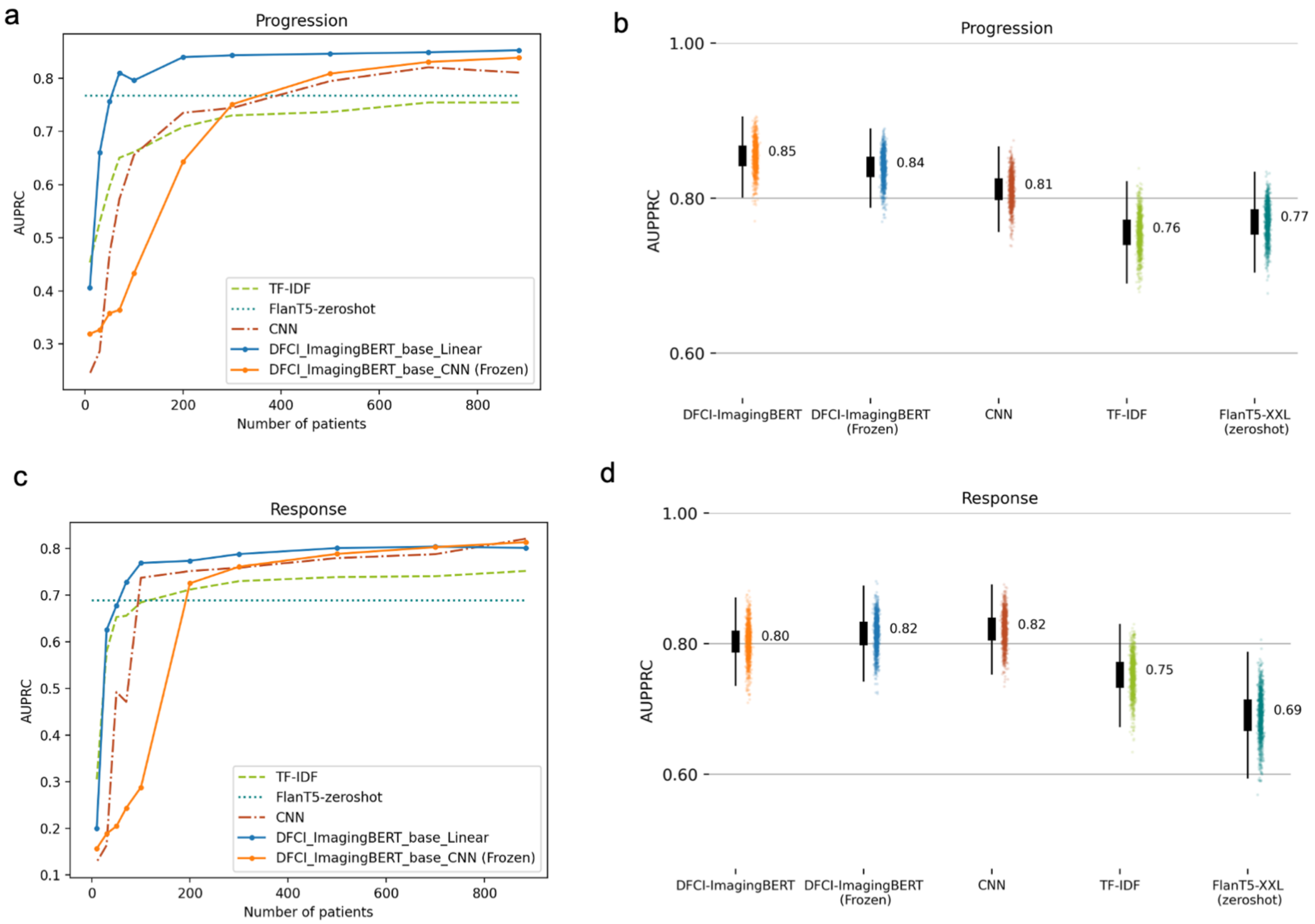

Supplement: Supplementary file 1 — Additional file 1: Supplementary Figures. [file 12859_2023_5439_MOESM1_ESM.pdf]
